# Using multitask classification methods to investigate the kinase-specific phosphorylation sites

**DOI:** 10.1186/1477-5956-10-S1-S7

**Published:** 2012-06-21

**Authors:** Shan Gao, Shuo Xu, Yaping Fang, Jianwen Fang

**Affiliations:** 1 Applied Bioinformatics Laboratory, Kansas University, 2034 Becker Dr., Lawrence, KS 66047, USA; 2 Institute of Scientific and Technical Information of China, No. 15 Fuxing Road, Haidian District, Beijing 100038, P.R. China

## Abstract

**Background:**

Identification of phosphorylation sites by computational methods is becoming increasingly important because it reduces labor-intensive and costly experiments and can improve our understanding of the common properties and underlying mechanisms of protein phosphorylation.

**Methods:**

A multitask learning framework for learning four kinase families simultaneously, instead of studying each kinase family of phosphorylation sites separately, is presented in the study. The framework includes two multitask classification methods: the Multi-Task Least Squares Support Vector Machines (MTLS-SVMs) and the Multi-Task Feature Selection (MT-Feat3).

**Results:**

Using the multitask learning framework, we successfully identify 18 common features shared by four kinase families of phosphorylation sites. The reliability of selected features is demonstrated by the consistent performance in two multi-task learning methods.

**Conclusions:**

The selected features can be used to build efficient multitask classifiers with good performance, suggesting they are important to protein phosphorylation across 4 kinase families.

## Background

Protein phosphorylation, one of the most important forms of post-translational modification of proteins, occurs on several different types of amino acid substrates. Serine (S) phosphorylation is the most common, followed by threonine (T) and tyrosine (Y). Histidine and aspartate phosphorylation may also occur, but mostly in prokaryotes as part of two-component signalling transduction systems [1] or rarely in some eukaryotic signal transduction pathways [2].

Protein kinases, which catalyze phosphorylation, play critical roles in the regulation of the majority of cellular pathways, including metabolism, signal transduction, transcription, translation, cell growth, and cell differentiation. Protein kinases account for approximately 2% of known human proteins, but they are responsible of phosphorylating approximate 30% of known human proteins [3]. Moreover, nearly half of human kinases are located in disease loci (such as asthma and autoimmunity) or cancer amplicons [4]. All protein kinases are often classified into several categories based on their substrate specificity. Serine/threonine (S/T) kinases, the most common category, are further classified into a number of kinase families, including cyclin-dependent kinase (CDK), casein kinase 2 (CK2), protein kinase A (PKA), and protein kinase C (PKC).

In recent years, identification of phosphorylation sites by computational methods is becoming increasingly important, with the growing gap between protein sequences information and annotated phosphorylation information of proteins with known sequences. That is due to still lack of high throughput experimental methods for identifying the phosphorylation sites of proteins and current technologies are labor-intensive and costly. Besides predicting phosphorylation sites, computational approaches can also be used to discover the common and specific features of different kinase groups.

A large number of computational tools for predicting phosphorylation sites have been reported [5]. These methods can be roughly grouped into two categories: kinase-specific predictors (e.g. Scansite [6], PredPhospho [7], PHOSITE [8], NetPhosK [9], GPS[10], KinasePhos [11], PPSP [12]) and non-specific predictors (e.g. NetPhos [13], DISPHOS [14]). Given a protein sequence, the non-specific methods can only predict whether a candidate site is a phosphorylation site or not, while kinase-specific methods can not only predict whether it is a phosphorylation site but also assign it to a specific kinase or a specific kinase family. Recently Ji et al. assessed 15 predictors and combined them to build a meta-predictor method named MetaPred [3]. The performance of MetaPred exceeded that of all these 15 member predictors in predicting kinase-specific phosphorylation sites across 4 kinase families. Like all meta-predictors, however, the performance of MetaPred depends on its member primary predictors. Moreover, it is impossible to evaluate the importance of individual features since different primary predictors use different sets of features.

All current kinase-specific phosphorylation prediction methods are single-task learning methods (STL) because they are trained independent from each other. Such methods are optimized on individual training datasets and thus the commonalities between different datasets are not considered. In this study, we use Multi-Task Learning (MTL) methods, instead of STL methods in previous studies, to investigate the kinase-specific phosphorylation sites by learning all STs simultaneously. Using a shared representation, MTL learns all participated STs of a problem by a global optimization approach based on an intuitive idea: the common knowledge shared by related STs in a specific domain helps improving the performance [15]. It has been empirically and theoretically demonstrated that MTL can improve learning performance, compared to learning STs separately [16]. In addition, MTL can be used to find the common knowledge and perform feature selection to identify significant features shared by member STs. MTL is particularly suitable for learning many STs with scarce data [17], which is currently considered as a major problem in the bioinformatics field. Recently, MTL has been successfully applied to study several biological problems, such as gene expression analysis [18], subcellular location of proteins [19], and prediction of siRNA efficacy [20].

In this study, we apply two MTL methods, namely the Multi-Task Least Squares Support Vector Machines (MTLS-SVMs) and the Multi-Task Feature Selection (MT-Feat3) to the data of 4 kinase families with phosphorylation sites using datasets collected by Ji et al [3]. MT-Feat3 is used to efficiently select features and MTLS-SVMs is then used to build classifiers to do cross validation.

As results, we identify 18 non-redundant common features, which are deemed as important to protein phosphorylation across 4 kinase families. Compared to the initial set of 560 features, the number of features used in the new predictor is reduced by more than 96% without deteriorating the performance. Based on those selected features, future work can be done to reveal some common mechanisms of phosphorylation by different kinase groups.

## Methods

### Dataset

The dataset MetaPS06 used in this study was downloaded [3]. It consists of 4 kinase family datasets including CDK, CK2, PKA, and PKC. For each kinase family dataset, positive samples are known phosphorylation sites, identified by experiments and belong to that family, while negative samples are non-phosphorylation sites or phosphorylation sites belonging to other families. Furthermore, multi-kinases phosphorylation sites were excluded in all datasets [3]. The numbers of positives/negatives in the final kinase family datasets are 294/441 (CDK), 229/343 (CK2), 360/540 (PKA), and 348/522(PKC).

### Feature extraction and peptide encoding

In this study, we use 560 features (physicochemical properties) of twenty amino acid residues. Among them, 544 features were obtained from AAindex database [21] and the remaining 16 features were collected from published literatures. All features are normalized to a range from 0 to 1.

A fixed length window is applied to scan a peptide sequence. The window size is optimized using odd numbers from 3 to 21. The average of features of all amino acids in a fixed window is assigned to the middle amino acid of the window. Thus the *i*th peptide is represented by N features in the form ${\vec{x}}_{i}=\left(x_{i1},x_{i2},\ldots x_{ij}\ldots x_{iN}\right)$, where N is 560.

### SVMs, RF and LS-SVMs

Support vector machines (SVMs) derive parameters of the maximum-margin to construct an optimized separating hyperplane. The optimization of SVM classifiers includes the selection of kernel, optimization of the kernel's parameters and soft margin parameter C.

Random Forest (RF) is an ensemble machine learning method that utilizes many independent decision trees to perform classification or regression. Each of member trees is built on a bootstrap sample from the training data by a random subset of available variables.

LS-SVMs can be considered as a variant of classical SVMs. LS-SVMs realize the optimization by solving a set of linear equations instead of a convex quadratic programming for SVMs. LS-SVMs perform training faster than SVMs without sacrificing generalization performance [22]. The LS-SVMs classifier is obtained by solving a restricted optimization problem as below (Formula 1).

(1) $$\begin{array}{c} \underset{w,e}{\min}\;\frac{1}{2}{∥\vec{w}∥}^{2}+\frac{1}{2}\gamma \overset{N}{\underset{i=1}{\sum }}{e_{i}}^{2}\\ \text{s}\text{.t}\text{.}\;y_{i}\left[<\vec{w},\phi \left({\vec{x}}_{i}\right)>+b\right]=1-e_{i},\;i=1,2,\ldots N \end{array}$$

where ${\vec{x}}_{i}$ is the sample, *y_i _*is its corresponding label, *N *is the sample number, *e_i _*is the error, $\vec{w}$ is the vector of weights, *ϕ*() is the non-linear mapping function, *γ *and b are parameters to be fitted.

### MTLS-SVMs

MTLS-SVMs is developed based on the mechanism of data amplification. An MTLS-SVMs classifier learns common parameters by integrating the sub datasets. It is obtained by solving a restricted optimization problem as below (Formula 2), and then the optimization problem can also be solved by solving linear equations.

(2) $$\begin{array}{l} \underset{w,e}{\min}\;\frac{1}{2}{\left\|{\vec{w}}_{0}\right\|}^{2}+\frac{1}{2}\frac{\lambda }{T}\sum_{t=1}^{T}{\vec{w}}_{t}^{T}{\vec{w}}_{t}+\frac{1}{2}\gamma \sum_{t=1}^{T}{\left\|{\vec{e}}_{t}\right\|}^{2}\\ s.t\;y_{ti}[<{\vec{w}}_{0}+{\vec{w}}_{t},\phi ({\vec{x}}_{ti})>+b_{t}]=1-e_{ti},i\in N_{t},t=1,2,\cdots T \end{array}$$

where *T *is the task number, *N_t _*is the sample number of the t^th ^task, ${\vec{w}}_{0}$ is the common weights shared by T single tasks, ${\vec{w}}_{t}$ is the weights for the t^th ^task, ${\vec{x}}_{ti}$ is the i^th ^sample of the t^th ^task, *y_ti _*is its corresponding label, *ϕ*() is the non-linear mapping function, *λ*, *γ *and b are parameters to be fitted.

### MT-Feat3

MT-Feats (Multi-Task Feature Learning and Selection) algorithm was derived from a MTL framework, which was designed to learn sparse representation shared cross STs from the training data [23]. MT-Feats algorithm originally includes two algorithms to solve the regression problems. The first one was developed for feature learning and the second was for feature selection.

We modify MT-Feats algorithms to solve classification problems, by using LS-SVMs as element classifiers. MT-Feat1 was developed for feature learning and MT-Feat3 was for feature selection. Both feature learning and feature selection learn common parameters by jointly regularizing a common term (Formula 3).

(3) $$\begin{array}{c} \underset{A,U}{\min}\;\frac{1}{2}\gamma \overset{T}{\underset{t=1}{\sum }}<{\vec{w}}_{t},D^{-1}{\vec{w}}_{t}>+\overset{T}{\underset{t=1}{\sum }}\overset{N_{t}}{\underset{i=1}{\sum }}\frac{1}{2T}{e_{ti}}^{2}\\ \text{s}\text{.t}\text{.}\;y_{ti}\left[<{\vec{w}}_{t},\phi \left({\vec{x}}_{ti}\right)>b_{t}\right]=1-e_{ti},i\in N_{t},t=1,2,\cdots T \end{array}$$

Where *W *= *UA*, other symbols have the same meaning as those in formula 2. If the U is set as identity matrix, the "Feature learning" problem (MT-Feat1) is reduced to a "Feature selection" problem (MT-Feat3). Thus, MT-Feat3 is a special case of MT-Feat1 algorithm (See Formula 4). In this study, we only use MT-Feat3 for feature selection.

(4) $$\begin{array}{c} \underset{A,U}{\min}\;\overset{T}{\underset{t=1}{\sum }}\overset{N_{t}}{\underset{i=1}{\sum }}L\left(y_{ti},<{\vec{a}}_{t},U^{T}{\vec{x}}_{ti}>\right)+\gamma {∥A∥}_{2,1}^{2}\\ \text{s}\text{.t}\text{.}\;U\in O^{D},A\in R^{D\times T} \end{array}$$

### Performance measures

Performance is measured by average accuracy (aveAc) which is described in formula 5.

(5) $$aveAc=\frac{TP+TN}{TP+TN+FP+FN}$$

Where TP and TN denote the total number of correctly classified positive and negative samples across all the STs. FP and FN denote the total number of incorrect classified positive and negative samples across all the STs. Since the datasets are relatively balanced, the average accuracy is sufficient to measure the performance of various predictors.

## Results

### Classification of family-specific phosphorylation sites by two MTL methods

We use MTLS-SVMs and MT-Feat3 methods to build classifiers for predicting phosphorylation sites on 4 kinase family datasets. To compare the performance of MTLS-SVMs and MT-Feat3 methods with that of the STL method, LS-SVMs classifiers are also built using the save datasets. Five-fold cross validation and grid-fitting of parameters are used to estimate the performance of all classifiers with window size from 3 to 21 (Table 1). It can be seen in Table 1 that in general there is an agreement on the average classification accuracy (aveAc) of all three methods on different window sizes and the window size of 7 delivers the best performance for both STL and MTL classifiers. However, apparently the performance of MTL methods (MTLS-SVMs and MT-Feat3) is inferior to the STL (LS-SVM) method. We hypothesize that a uniform window size may be not a good choice for all four kinase family datasets because of the specificity of each kinase. Secondly, there are many redundant or irrelevant features that may decrease the performance. Therefore, in the following work we attempt to improve the performance of MTL classifiers by optimizing window sizes and performing feature selection.

**Table 1:** Average classification accuracy of different classifiers with 560 features

window size	LS-SVMs	MTL-Feat3	MTLS-SVMs
3	0.7381	0.727	0.728
5	0.754	0.7462	0.7459
7	0.7611	0.7595	0.7595
9	0.7498	0.741	0.74
11	0.7504	0.7455	0.7478
13	0.7491	0.7403	0.7416
15	0.7439	0.7355	0.7394
17	0.7439	0.729	0.7316
19	0.7325	0.7251	0.727
21	0.7325	0.7192	0.7176
opt*	0.7939	0.791	0.7936

Five fold cross validation and grid fitting of parameters are used to estimate the performance of all classifiers. *The optimized window sizes (3, 17, 7 and 9) for 4 kinase family datasets are used to build classifiers.

### Optimized window sizes for 4 kinase family

For local window based methods, a proper window size reflects the optimized physical or chemical effects on the central amino acid from local surroundings. Different window sizes have been used in previous studies. For example, GPS [10], KinasePhos [11], PPSP [12] used a symmetrical window of 7 consecutive amino acid residues (7-mer), and NetPhosK [9] used 15-mer and 17 mer. Instead of assuming a uniform window size for all kinase families, we build classifiers based on Support Vector Machines (SVMs) and Random Forest (RF) algorithms to optimize the window size for each of the kinase family dataset. We use ten-fold cross validation and grid fitting of parameters to estimate the performance of all classifiers with 560 features (Table 2). The results clearly show that the performance of both SVMs and RF has very similarly tendency for different window sizes and optimized window sizes are insensitive to the classification algorithms. Generally, SVM models using the linear kernel deliver better performance than SVM models with the rbf kernel and RF models. Using the optimized window sizes respectively presented in Table 2 (3, 17, 7 and 9 for CDK, CK2, PKA and PKC datasets), we build respective models and compare the results with the models using uniform window sizes (Table 1). It is clear that the optimized window sizes significantly improve the performance of LS-SVMs (aveAc = 0.7939), MTLS-SVMs (aveAc = 0.7936), and MT-Feat3 (aveAc = 0.791). In the following parts, window sizes with 3, 17, 7 and 9 for CDK, CK2, PKA and PKC datasets respectively are referred as optimized window sizes.

**Table 2:** Classification accuracy of different classifiers with 560 features for 4 kinase datasets

	CDK kinase family			CK2 kinase family			PKA kinase family			PKC kinase family		
window size	SVM-rbf	SVM-linear	RF	SVM -rbf	SVM-linear	RF	SVM-rbf	SVM-linear	RF	SVM-rbf	SVM-linear	RF
3	0.8598	0.8613*	0.83	0.7783	0.7796	0.7326	0.6656	0.6678	0.6289	0.6723	0.6758	0.6113
5	0.8013	0.8122	0.7579	0.806	0.8112	0.7935	0.7156	0.7178	0.7111	0.7173	0.724	0.7069
7	0.7578	0.7581	0.7455	0.8655	0.8724	0.8599	0.7567	0.7533*	0.7589	0.7242	0.7196	0.7253
9	0.7305	0.7077	0.7171	0.8706	0.8688	0.8548	0.7456	0.7489	0.7622	0.7253	0.7393*	0.7183
11	0.7223	0.724	0.7226	0.8654	0.8617	0.8619	0.7433	0.7478	0.7511	0.7161	0.7298	0.7023
13	0.721	0.7103	0.7049	0.867	0.8705	0.874	0.7367	0.7378	0.7311	0.7287	0.7299	0.7299
15	0.7211	0.7023	0.7049	0.8724	0.8723	0.8792	0.7322	0.7267	0.7156	0.7264	0.7286	0.7253
17	0.7087	0.7038	0.717	0.8812	0.8811*	0.874	0.7211	0.7167	0.7089	0.7253	0.7286	0.7252
19	0.7142	0.6995	0.7049	0.8759	0.8844	0.8757	0.72	0.6978	0.7167	0.7173	0.7194	0.7194
21	0.7263	0.6887	0.72	0.8759	0.8775	0.8739	0.7133	0.7022	0.7044	0.7082	0.7309	0.6999

*Best performance for each kinase family by SVM with linear kernel and the corresponding window size is selected as the final optimized window size for that kinase family.

### Feature selection and validation

Feature selection can improve the performance of classifiers not only in delivering faster and more effective classifiers but also in providing better understanding of relevant biological processes. MT-Feat3 is capable of selecting common features across multi tasks in addition to performing classification. We firstly construct a weight matrix W with a dimension of 560*4 to represent the significance of 560 features across 4 kinase family datasets using a uniform windows size of 7. The MT-Feat3 can significantly reduce the dimension of features by eliminating rows with zero weights. We then compute the 2-norm weight $w_{i}=\sqrt{{\overset{4}{\underset{j=1}{\sum }}W_{ij}}^{2}}$ of each non-zero row in W and obtain the significance *w_i _*which represents the importance of the ith feature among 4 kinase family datasets. All non-zero features with *w_i _*^2 ^larger than zero are considered as significant common features and their importance is sorted accordingly. In addition, the same procedure of feature selection is conducted using the optimized window sizes for 4 kinase family datasets (Table 2).

**Figure 1:**
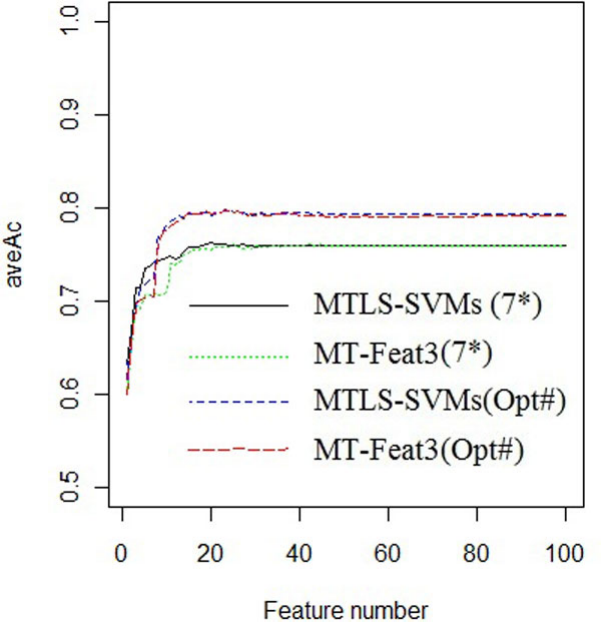
**Performance with different feature numbers**. *window size 7 across 4 kinase family datasets. # optimized window sizes (3, 17, 7 and 9) across 4 kinase family datasets.

Using various numbers of the most important features, ranked by the models using either the uniform window or optimized windows, we develop two series of MT-Feat3 models accordingly. In addition, we develop corresponding MTLS-SVM classifiers using the same sets of features. The average accuracies of all models are displayed in Figure 1. Based on the Figure 1, we select 20 features for the models using the window size of 7 and 26 features for the models using optimized window size. The MTLS-SVM models using these sets of features achieve average accuracies of 0.7621 and 0.7962, higher than that (aveAc with 0.7595 and 0.7936) of MTLS-SVMs before feature selection (Table 3). Thus it is clear that feature selection by MT-Feat3 can improve the performance and the performance of MT-Feat3 and MTLS-SVMs is quite consistent. In addition, using optimized windows results in better performance than using a uniform window size of 7 (Table 3). The performance of MTLS-SVM model using the 26 selected features with the optimized window sizes achieves comparable performance to MetaPred (0.7962 vs 0.7997).

**Table 3:** Classification Accuracy of different classifiers with selected features

Methods	Window size	Feature number	aveAc
MetaPred	NA	NA	**0.7997**
LS-SVMs	7	560	0.7611
	opt	560	0.7939
*MT-Feat3	7	25	0.7605
	opt	23	0.7972
MTLS-SVMs	7	560	0.7595
	opt	560	0.7936
*MTLS-SVMs	7	20	0.7621
	opt	26	**0.7962**
#MTLS-SVMs	7	12	0.7455
	opt	18	**0.792**

Five fold cross validation and grid fitting of parameters are used to estimate the performance of all the classifiers.

*Using features selected by the MT-Feat3 method.

#Using features filtered by the Metric multi-dimensional scaling method.

### Analysis of selected features

The selected features subset 1 (20 features) and subset 2 (26 features) using the uniform window size of 7 or the optimized window sizes, respectively, are listed in Table 4. There are 14 common features appear in both subset 1 and subset 2. These common 14 features can be grouped into 6 categories, including backbone electrostatic interactions ("AVBF000101", "AVBF000102", "AVBF000104", "AVBF000105", "AVBF000106", "AVBF000107", "AVBF000108", "AVBF000109"), hydrophobicity ("ROSM880104", "ROSM880105"), apparent partition energies ("GUYH850103"), negative charge ("FAUJ880112"), fractional occurrence in left helix regions ("RACS820103") and side chain conformation ("YANJ020101").

**Table 4:** Selected features by MT-Feat3

	Subset 1 (20 features)	Subset 2 (26 features)
Backbone electrostatic interactions	AVBF000101*#	AVBF000101*#
	AVBF000102*#	AVBF000102*#
	AVBF000104*#	AVBF000104*#
	AVBF000105*#	AVBF000105*#
	AVBF000106*	AVBF000106*
	AVBF000107*#	AVBF000107*#
	AVBF000108*#	AVBF000108*#
	AVBF000109*	AVBF000109*#
Hydrophobicity	ROSM880104*	ROSM880104*
	ROSM880105*#	ROSM880105*
Apparent partition energies	GUYH850103*	GUYH850103*
Negative charge	FAUJ880112*	FAUJ880112*
Fractional occurrence in left helix regions	RACS820103*	RACS820103*
Side chain conformation others	YANJ020101*	YANJ020101*
	CHAM830108	SNEP660101
	PALJ810113	BUNA790103
	WILM950104	CRAJ730101
	BURA740101	TANS770102
	JOND920102	BULH740101
	AVBF000103#	GEIM800103
		PALJ810107
		GEIM800105
		VELV850101
		COSI940101#
		ISOY800107
		CHOP780211

Features in Subset 1 are selected by MT-Feat3 with window size 7. Features in Subset 2 are selected by MT-Feat3 with optimized window sizes across 4 kinase family datasets.

*Common features shared by subset 1 (20 features) and subset 2 (26 features).

# Features filtered by the Metric multi-dimensional scaling method.

**Figure 2:**
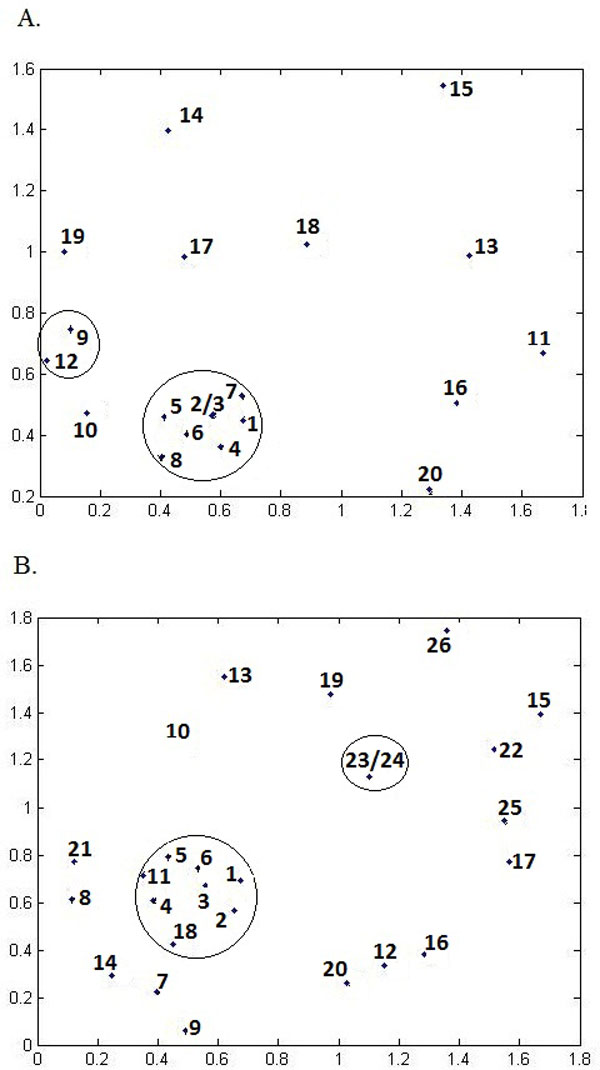
**Two-dimensional map by metric multi-dimensional scaling method**. (A) Subset 1 selected by MT-Feat3 with window size 7. Redundant features (in circles) are removed, leading to subset 3. (B) Subset 2 selected by MT-Feat3 with optimized window sizes. Redundant features (in circles) are removed, leading to feature subset 4. All the removed features are marked # in Table 4.

To investigate the relationship between selected features, we cluster features in the subset 1 (Figure 2A) and subset 2 (Figure 2B) by Pearson correlation coefficients distances and constructed a two-dimensional map (Figure 2A) by the metric multi-dimensional scaling method [24]. All features with high correlation coefficients with other features (labelled by # in Table 4) are removed from the subset 1 and 2 respectively, resulted in the subset 3 (12 features) and subset 4 (18 features). The detailed description of the subsets 1, 2, 3 and 4 is available in Additional file 1.

The best aveAc of MTLS-SVMs with the subset 4 is 0.792, very close to that of MTLS-SVMs with total features (0.7936). The best aveAc of MTLS-SVMs with the subset 3 is 0.7455, which is slightly poorer than that of MTLS-SVMs with total features (0.7595) (Table 4). Therefore, those 18 features in subset 4 are considered as significant properties related with protein phosphorylation.

## Conclusions

In this study, we use a multi-task learning framework to investigate phosphorylation sites across 4 kinase family datasets. In this framework, MT-Feat3 is used to select some common features, which are then validated by MTLS-SVMs classifiers. Selected features are further reduced to 18 features after eliminating features with high correlation coefficients with outer features. These features are considered as important common features for further analysis of possible properties and mechanisms of protein phosphorylation.

## Competing interests

The authors declare that they have no competing interests.

## Authors' contributions

SG and SX developed the programs. SG and YF did the dataset construction and calculation, and drafted the manuscript. JF conceived of the project, and participated in its design and coordination and helped to draft the manuscript. All authors read and approved the final manuscript.

## Supplementary Material

Additional file 1 **Description of selected features from AAIndex**. Descriptions of AAIndex records corresponding to selected features in subset 1, 2, 3 and 4. Click here for file
