# Supplementary material for: Using multitask classification methods to investigate the kinase-specific phosphorylation sites
Source: Proteome Sci. 2012 Jun 21;10(Suppl 1):S7. doi: 10.1186/1477-5956-10-S1-S7 (PMC3380725; doi:10.1186/1477-5956-10-S1-S7)
Supplement: Additional file 1 — Description of selected features from AAIndex. Descriptions of AAIndex records corresponding to selected features in subset 1, 2, 3 and 4. [file 1477-5956-10-S1-S7-S1.pdf]

Description of selected features from AAIndex

Selected features (20) in subset 1

AVBF000101
AVBF000102
AVBF000104
AVBF000105
AVBF000106
AVBF000107
AVBF000108
AVBF000109
ROSM880104
ROSM880105
GUYH850103
FAUJ880112
RACS820103
YANJ020101
CHAM830108
PALJ810113
WILM950104
BURA740101
JOND920102
AVBF000103

Selected features (26) in subset 2

AVBF000101*#
AVBF000102*#
AVBF000104*#
AVBF000105*#
AVBF000106*
AVBF000107*#
AVBF000108*#
AVBF000109*#
ROSM880104*
ROSM880105*
GUYH850103*
FAUJ880112*
RACS820103*
YANJ020101*
SNEP660101
BUNA790103
CRAJ730101
TANS770102
BULH740101
GEIM800103
PALJ810107
GEIM800105
VELV850101
COSI940101#
ISOY800107
CHOP780211

Features (12) of subset 3

AVBF000106
AVBF000109
ROSM880104
GUYH850103
FAUJ880112
RACS820103
YANJ020101
CHAM830108
PALJ810113
WILM950104
BURA740101
JOND920102

Features (18) of subset 3

AVBF000106*
ROSM880104
ROSM880105
GUYH850103
FAUJ880112
RACS820103
YANJ020101
SNEP660101
BUNA790103
CRAJ730101
TANS770102
BULH740101
GEIM800103
PALJ810107
GEIM800105
VELV850101
ISOY800107
CHOP780211

AAindex database records

//
H AVBF000106
D Slopes tripeptide FDPB VFF all (Avbelj, 2000)
R PMID:10903873
A Avbelj, F.
T Amino acid conformational preferences and solvation of polar backbone atoms
in peptides and proteins
J J. Mol. Biol. 300, 1335-1359 (2000) (Pro missing)

I	A/L	R/K	N/M	D/F	C/P	Q/S	E/T	G/W	H/Y	I/V
	-0.378	-0.369	-0.245	-0.113	-0.206	-0.290	-0.165	-0.560	-0.295	-0.134
	-0.266	-0.335	-0.260	-0.187	NA	-0.251	-0.093	-0.188	-0.147	-0.084

//
H AVBF000105
D Slopes tripeptide, FDPB VFF noside (Avbelj, 2000)
R PMID:10903873
A Avbelj, F.
T Amino acid conformational preferences and solvation of polar backbone atoms
in peptides and proteins
J J. Mol. Biol. 300, 1335-1359 (2000) (Pro missing)

I	A/L	R/K	N/M	D/F	C/P	Q/S	E/T	G/W	H/Y	I/V

-0.393 -0.317 -0.268 -0.247 -0.222 -0.291 -0.260 -0.570 -0.244 -0.144
-0.281 -0.294 -0.274 -0.189 NA -0.280 -0.152 -0.206 -0.155 -0.080
//
H AVBF000107
D Slopes tripeptide FDPB PARSE neutral (Avbelj, 2000)
R PMID:10903873
A Avbelj, F.
T Amino acid conformational preferences and solvation of polar backbone atoms
in peptides and proteins
J J. Mol. Biol. 300, 1335-1359 (2000) (Pro missing)
I A/L R/K N/M D/F C/P Q/S E/T G/W H/Y I/V
-0.729 -0.535 -0.597 -0.545 -0.408 -0.492 -0.532 -0.860 -0.519 -0.361
-0.462 -0.508 -0.518 -0.454 NA -0.278 -0.367 -0.455 -0.439 -0.323
//
H AVBF000101
D Screening coefficients gamma, local (Avbelj, 2000)
R PMID:10903873
A Avbelj, F.
T Amino acid conformational preferences and solvation of polar backbone atoms
in peptides and proteins
J J. Mol. Biol. 300, 1335-1359 (2000) (Pro missing)
I A/L R/K N/M D/F C/P Q/S E/T G/W H/Y I/V
0.163 0.220 0.124 0.212 0.316 0.274 0.212 0.080 0.315 0.474
0.315 0.255 0.356 0.410 NA 0.290 0.412 0.325 0.354 0.515
//
H AVBF000104
D Slopes tripeptides, LD VFF neutral (Avbelj, 2000)
R PMID:10903873
A Avbelj, F.
T Amino acid conformational preferences and solvation of polar backbone atoms
in peptides and proteins
J J. Mol. Biol. 300, 1335-1359 (2000) (Pro missing)
I A/L R/K N/M D/F C/P Q/S E/T G/W H/Y I/V
-0.871 -0.727 -0.741 -0.737 -0.666 -0.728 -0.773 -0.822 -0.685 -0.617
-0.798 -0.715 -0.717 -0.649 NA -0.679 -0.629 -0.669 -0.655 -0.599
//
H AVBF000102
D Screening coefficients gamma, non-local (Avbelj, 2000)
R PMID:10903873
A Avbelj, F.
T Amino acid conformational preferences and solvation of polar backbone atoms
in peptides and proteins
J J. Mol. Biol. 300, 1335-1359 (2000) (Pro missing)
I A/L R/K N/M D/F C/P Q/S E/T G/W H/Y I/V
0.236 0.233 0.189 0.168 0.259 0.314 0.306 -0.170 0.256 0.391
0.293 0.231 0.367 0.328 NA 0.202 0.308 0.197 0.223 0.436
//
H AVBF000108
D Slopes dekapeptide, FDPB VFF neutral (Avbelj, 2000)
R PMID:10903873
A Avbelj, F.
T Amino acid conformational preferences and solvation of polar backbone atoms
in peptides and proteins
J J. Mol. Biol. 300, 1335-1359 (2000) (Pro missing)
I A/L R/K N/M D/F C/P Q/S E/T G/W H/Y I/V
-0.623 -0.567 -0.619 -0.626 -0.571 -0.559 -0.572 -0.679 -0.508 -0.199
-0.527 -0.581 -0.571 -0.461 NA -0.458 -0.233 -0.327 -0.451 -0.263
//
H ROSM880104

D Hydropathies of amino acid side chains, neutral form (Roseman, 1988)
R PMID:3398047
A Roseman, M.A.
T Hydrophilicity of Polar Amino Acid Side-chains is Markedly Reduced by
Flanking Peptide Bonds
I A/L R/K N/M D/F C/P Q/S E/T G/W H/Y I/V
0.39 NA -1.91 -0.71 0.25 -1.30 -0.18 0.00 -0.60 1.82
1.82 0.32 0.96 2.27 NA -1.24 -1.00 2.13 1.47 1.30
//
H AVBF000109
D Slopes proteins, FDPB VFF neutral (Avbelj, 2000)
R PMID:10903873
A Avbelj, F.
T Amino acid conformational preferences and solvation of polar backbone atoms
in peptides and proteins
J J. Mol. Biol. 300, 1335-1359 (2000) (Pro missing)
I A/L R/K N/M D/F C/P Q/S E/T G/W H/Y I/V
-0.376 -0.280 -0.403 -0.405 -0.441 -0.362 -0.362 -0.392 -0.345 -0.194
-0.317 -0.412 -0.312 -0.237 NA -0.374 -0.243 -0.111 -0.171 -0.355
//
H GUYH850103
D Apparent partition energies calculated from Robson-Osguthorpe index (Guy,
1985)
R PMID:3978191
A Guy, H.R.
T Amino acid side-chain partition energies and distribution of residues in
soluble proteins
J Biophys. J. 47, 61-70 (1985) (Gly missing)
I A/L R/K N/M D/F C/P Q/S E/T G/W H/Y I/V
0.54 -0.16 0.38 0.65 -1.13 0.05 0.38 NA -0.59 -2.15
-1.08 0.48 -0.97 -1.51 -0.22 0.65 0.27 -1.61 -1.13 -0.75
//
H ROSM880105
D Hydropathies of amino acid side chains, pi-values in pH 7.0 (Roseman, 1988)
R PMID:3398047
A Roseman, M.A.
T Hydrophilicity of Polar Amino Acid Side-chains is Markedly Reduced by
Flanking Peptide Bonds
J J. Mol. Biol. 200, 513-522 (1988) (Pro missing)
I A/L R/K N/M D/F C/P Q/S E/T G/W H/Y I/V
0.39 -3.95 -1.91 -3.81 0.25 -1.30 -2.91 0.00 -0.64 1.82
1.82 -2.77 0.96 2.27 NA -1.24 -1.00 2.13 1.47 1.30
//
H FAUJ880112
D Negative charge (Fauchere et al., 1988)
R LIT:1414114 PMID:3209351
A Fauchere, J.L., Charton, M., Kier, L.B., Verloop, A. and Pliska, V.
T Amino acid side chain parameters for correlation studies in biology and
pharmacology
J Int. J. Peptide Protein Res. 32, 269-278 (1988)
I A/L R/K N/M D/F C/P Q/S E/T G/W H/Y I/V
0. 0. 0. 1. 0. 0. 1. 0. 0. 0.
0. 0. 0. 0. 0. 0. 0. 0. 0. 0.
//
H RACS820103
D Average relative fractional occurrence in AL(i) (Rackovsky-Scheraga, 1982)
R LIT:0903736
A Rackovsky, S. and Scheraga, H.A.
T Differential geometry and polymer conformation. 4. Conformational and

nucleation properties of individual amino acids
J Macromolecules 15, 1340-1346 (1982)

I	A/L	R/K	N/M	D/F	C/P	Q/S	E/T	G/W	H/Y	I/V
	0.82	2.60	2.07	2.64	0.00	0.00	2.62	1.63	0.00	2.32
	0.00	2.86	0.00	0.00	0.00	1.23	2.48	0.00	1.90	1.62

//
H YANJ020101
D Side-chain conformation by gaussian evolutionary method (Yang et al., 2002)
R PMID:12142444
A Yang, J.M., Tsai, C.H., Hwang, M.J., Tsai, H.K., Hwang, J.K. and Kao, C.Y.
T GEM: a Gaussian Evolutionary Method for predicting protein side-chain conformations
J Protein Sci. 11, 1897-1907 (2002) (Gly Ala missing)

I	A/L	R/K	N/M	D/F	C/P	Q/S	E/T	G/W	H/Y	I/V
	NA	0.62	0.76	0.66	0.83	0.59	0.73	NA	0.92	0.88
	0.89	0.77	0.77	0.92	0.94	0.58	0.73	0.86	0.93	0.88

//
H AVBF000103
D Slopes tripeptide, FDPB VFF neutral (Avbelj, 2000)
R PMID:10903873
A Avbelj, F.
T Amino acid conformational preferences and solvation of polar backbone atoms in peptides and proteins
J J. Mol. Biol. 300, 1335-1359 (2000) (Pro missing)

I	A/L	R/K	N/M	D/F	C/P	Q/S	E/T	G/W	H/Y	I/V
	-0.490	-0.429	-0.387	-0.375	-0.352	-0.422	-0.382	-0.647	-0.357	-0.268
	-0.450	-0.409	-0.375	-0.309	NA	-0.426	-0.240	-0.325	-0.288	-0.220

//
H CHAM830108
D A parameter of charge transfer donor capability (Charton-Charton, 1983)
R LIT:0907093b PMID:6876837
A Charton, M. and Charton, B.
T The dependence of the Chou-Fasman parameters on amino acid side chain structure
J J. Theor. Biol. 111, 447-450 (1983) (Pro !)

I	A/L	R/K	N/M	D/F	C/P	Q/S	E/T	G/W	H/Y	I/V
	0.	1.	1.	0.	1.	1.	0.	0.	1.	0.
	0.	1.	1.	1.	0.	0.	0.	1.	1.	0.

//
H PALJ810113
D Normalized frequency of turn in all-alpha class (Palau et al., 1981)
R LIT:0805095 PMID:7118409
A Palau, J., Argos, P. and Puigdomenech, P.
T Protein secondary structure
J Int. J. Peptide Protein Res. 19, 394-401 (1981) LG :a set of protein samples formed by 44 proteins. CF :a set of protein samples formed by 33 proteins. (Arg Cys Leu Trp missing)

I	A/L	R/K	N/M	D/F	C/P	Q/S	E/T	G/W	H/Y	I/V
	0.69	0.	1.52	2.42	0.	1.44	0.63	2.64	0.22	0.43
	0.	1.18	0.88	2.20	1.34	1.43	0.28	0.	1.53	0.14

//
H WILM950104
D Hydrophobicity coefficient in RP-HPLC, C18 with 0.1%TFA/2-PrOH/MeCN/H2O (Wilce et al. 1995)
R
A Wilce, M.C., Aguilar, M.I. and Hearn, M.T.
T Physicochemical basis of amino acid hydrophobicity scales: evaluation of four new scales of amino acid hydrophobicity coefficients derived from RP-HPLC of peptides

J Anal Chem. 67, 1210-1219 (1995)
I A/L R/K N/M D/F C/P Q/S E/T G/W H/Y I/V
-2.34 1.60 2.81 -0.48 5.03 0.16 1.30 -1.06 -3.00 7.26
1.09 1.56 0.62 2.57 -0.15 1.93 0.19 3.59 -2.58 2.06
//
H BURA740101
D Normalized frequency of alpha-helix (Burgess et al., 1974)
R LIT:2004075b
A Burgess, A.W., Ponnuswamy, P.K. and Scheraga, H.A.
T Analysis of conformations of amino acid residues and prediction of backbone
topography in proteins
J Isr. J. Chem. 12, 239-286 (1974)
I A/L R/K N/M D/F C/P Q/S E/T G/W H/Y I/V
0.486 0.262 0.193 0.288 0.200 0.418 0.538 0.120 0.400 0.370
0.420 0.402 0.417 0.318 0.208 0.200 0.272 0.462 0.161 0.379
//
H JOND920102
D Relative mutability (Jones et al., 1992)
R LIT:1814076 PMID:1633570
A Jones, D.T., Taylor, W.R. and Thornton, J.M.
T The rapid generation of mutation data matrices from protein sequences
J CABIOS 8, 275-282 (1992)
I A/L R/K N/M D/F C/P Q/S E/T G/W H/Y I/V
100. 83. 104. 86. 44. 84. 77. 50. 91. 103.
54. 72. 93. 51. 58. 117. 107. 25. 50. 98.
//
H SNEP660101
D Principal component I (Sneath, 1966)
R PMID:4291386
A Sneath, P.H.A.
T Relations between chemical structure and biological activity in peptides
J J. Theor. Biol. 12, 157-195 (1966)
I A/L R/K N/M D/F C/P Q/S E/T G/W H/Y I/V
0.239 0.211 0.249 0.171 0.220 0.260 0.187 0.160 0.205 0.273
0.281 0.228 0.253 0.234 0.165 0.236 0.213 0.183 0.193 0.255
//
H BUNA790103
D Spin-spin coupling constants 3JHalpha-NH (Bundi-Wuthrich, 1979)
R LIT:0503064b
A Bundi, A. and Wuthrich, K.
T 1H-nmr parameters of the common amino acid residues measured in aqueous
solutions of the linear tetrapeptides H-Gly-Gly-X-L-Ala-OH
J Biopolymers 18, 285-297 (1979) (Met Pro Trp !)
I A/L R/K N/M D/F C/P Q/S E/T G/W H/Y I/V
6.5 6.9 7.5 7.0 7.7 6.0 7.0 5.6 8.0 7.0
6.5 6.5 0. 9.4 0. 6.5 6.9 0. 6.8 7.0
//
H CRAJ730101
D Normalized frequency of middle helix (Crawford et al., 1973)
R PMID:4510294
A Crawford, J.L., Lipscomb, W.N. and Schellman, C.G.
T The reverse turn as a polypeptide conformation in globular proteins
J Proc. Natl. Acad. Sci. USA 70, 538-542 (1973) Reported values normalized by
the total percentage
I A/L R/K N/M D/F C/P Q/S E/T G/W H/Y I/V
1.33 0.79 0.72 0.97 0.93 1.42 1.66 0.58 1.49 0.99
1.29 1.03 1.40 1.15 0.49 0.83 0.94 1.33 0.49 0.96
//
H TANS770102

D Normalized frequency of isolated helix (Tanaka-Scheraga, 1977)
R PMID:557155
A Tanaka, S. and Scheraga, H.A.
T Statistical mechanical treatment of protein conformation. 5. A multiphasic
model for specific-sequence copolymers of amino acids
J Macromolecules 10, 9-20 (1977) Recalculated by Kidera as normalized
frequencies

I	A/L	R/K	N/M	D/F	C/P	Q/S	E/T	G/W	H/Y	I/V
	0.946	1.128	0.432	1.311	0.481	1.615	0.698	0.360	2.168	1.283
	1.192	1.203	0.000	0.963	2.093	0.523	1.961	1.925	0.802	0.409

//
H BULH740101
D Transfer free energy to surface (Bull-Breese, 1974)
R PMID:4839053
A Bull, H.B. and Breese, K.
T Surface tension of amino acid solutions: A hydrophobicity scale of the amino
acid residues
J Arch. Biochem. Biophys. 161, 665-670 (1974)

I	A/L	R/K	N/M	D/F	C/P	Q/S	E/T	G/W	H/Y	I/V
	-0.20	-0.12	0.08	-0.20	-0.45	0.16	-0.30	0.00	-0.12	-2.26
	-2.46	-0.35	-1.47	-2.33	-0.98	-0.39	-0.52	-2.01	-2.24	-1.56

//
H GEIM800103
D Alpha-helix indices for beta-proteins (Geisow-Roberts, 1980)
R LIT:0701087b
A Geisow, M.J. and Roberts, R.D.B.
T Amino acid preferences for secondary structure vary with protein class
J Int. J. Biol. Macromol. 2, 387-389 (1980)

I	A/L	R/K	N/M	D/F	C/P	Q/S	E/T	G/W	H/Y	I/V
	1.55	0.20	1.20	1.55	1.44	1.13	1.67	0.59	1.21	1.27
	1.25	1.20	1.37	0.40	0.21	1.01	0.55	1.86	1.08	0.64

//
H PALJ810107
D Normalized frequency of alpha-helix in all-alpha class (Palau et al., 1981)
R LIT:0805095 PMID:7118409
A Palau, J., Argos, P. and Puigdomenech, P.
T Protein secondary structure
J Int. J. Peptide Protein Res. 19, 394-401 (1981) LG :a set of protein samples
formed by 44 proteins. CF :a set of protein samples formed by 33 proteins.

I	A/L	R/K	N/M	D/F	C/P	Q/S	E/T	G/W	H/Y	I/V
	1.08	0.93	1.05	0.86	1.22	0.95	1.09	0.85	1.02	0.98
	1.04	1.01	1.11	0.96	0.91	0.95	1.15	1.17	0.80	1.03

//
H GEIM800105
D Beta-strand indices (Geisow-Roberts, 1980)
R LIT:0701087b
A Geisow, M.J. and Roberts, R.D.B.
T Amino acid preferences for secondary structure vary with protein class
J Int. J. Biol. Macromol. 2, 387-389 (1980)

I	A/L	R/K	N/M	D/F	C/P	Q/S	E/T	G/W	H/Y	I/V
	0.84	1.04	0.66	0.59	1.27	1.02	0.57	0.94	0.81	1.29
	1.10	0.86	0.88	1.15	0.80	1.05	1.20	1.15	1.39	1.56

//
H VELV850101
D Electron-ion interaction potential (Veljkovic et al., 1985)
R LIT:2004067b
A Veljkovic, V., Cosic, I., Dimitrijevic, B. and Lalovic, D.
T Is it possible to analyze DNA and protein sequences by the method of digital
signal processing?

J IEEE Trans. Biomed. Eng. 32, 337-341 (1985)
I A/L R/K N/M D/F C/P Q/S E/T G/W H/Y I/V
.03731 .09593 .00359 .12630 .08292 .07606 .00580 .00499 .02415 .00000
.00000 .03710 .08226 .09460 .01979 .08292 .09408 .05481 .05159 .00569
//
H COSI940101
D Electron-ion interaction potential values (Cosic, 1994)
R PMID:7851912
A Cosic, I.
T Macromolecular bioactivity: is it resonant interaction between macromolecules?--Theory and applications
J IEEE Trans Biomed Eng. 41, 1101-1114 (1994) (values are cited from Protein Eng. 15:193-203)
I A/L R/K N/M D/F C/P Q/S E/T G/W H/Y I/V
0.0373 0.0959 0.0036 0.1263 0.0829 0.0761 0.0058 0.0050 0.0242 0.0000
0.0000 0.0371 0.0823 0.0946 0.0198 0.0829 0.0941 0.0548 0.0516 0.0057
//
H ISOY800107
D Normalized relative frequency of double bend (Isogai et al., 1980)
R LIT:2004053b PMID:7378550
A Isogai, Y., Nemethy, G., Rackovsky, S., Leach, S.J. and Scheraga,H.A
T Characterization of multiple bends in proteins
J Biopolymers 19, 1183-1210 (1980) Recalculated by Kidera using a different set of proteins
I A/L R/K N/M D/F C/P Q/S E/T G/W H/Y I/V
1.34 2.78 0.92 1.77 1.44 0.79 2.54 0.95 0.00 0.52
1.05 0.79 0.00 0.43 0.37 0.87 1.14 1.79 0.73 0.00
//
H CHOP780211
D Normalized frequency of C-terminal non beta region (Chou-Fasman, 1978b)
R PMID:364941
A Chou, P.Y. and Fasman, G.D.
T Prediction of the secondary structure of proteins from their amino acid sequence
J Adv. Enzymol. 47, 45-148 (1978)
I A/L R/K N/M D/F C/P Q/S E/T G/W H/Y I/V
0.74 1.05 1.13 1.32 0.53 0.77 0.85 1.68 0.96 0.53
0.59 0.82 0.85 0.44 1.69 1.49 1.16 1.59 1.01 0.59
